# Omics and Multi-Omics Analysis for the Early Identification and Improved Outcome of Patients with Psoriatic Arthritis

**DOI:** 10.3390/biomedicines10102387

**Published:** 2022-09-24

**Authors:** Robert Gurke, Annika Bendes, John Bowes, Michaela Koehm, Richard M. Twyman, Anne Barton, Dirk Elewaut, Carl Goodyear, Lisa Hahnefeld, Rainer Hillenbrand, Ewan Hunter, Mark Ibberson, Vassilios Ioannidis, Sabine Kugler, Rik J. Lories, Eduard Resch, Stefan Rüping, Klaus Scholich, Jochen M. Schwenk, James C. Waddington, Phil Whitfield, Gerd Geisslinger, Oliver FitzGerald, Frank Behrens, Stephen R. Pennington, on behalf of the HIPPOCRATES Consortium

**Affiliations:** 1 Fraunhofer Institute for Translational Medicine and Pharmacology ITMP, Theodor-Stern-Kai 7, 60596 Frankfurt am Main, Germany; 2 Fraunhofer Cluster of Excellence Immune-Mediated Diseases CIMD, Theodor-Stern-Kai 7, 60596 Frankfurt am Main, Germany; 3 Pharmazentrum Frankfurt/ZAFES, Institute of Clinical Pharmacology, Goethe University, Theodor-Stern-Kai 7, 60590 Frankfurt am Main, Germany; 4 Science for Life Laboratory, School of Engineering Sciences in Chemistry, Biotechnology and Health, KTH Royal Institute of Technology, 171 65 Solna, Sweden; 5 NIHR Manchester Biomedical Research Centre, Manchester University NHS Foundation Trust, Manchester Academic Health Science Centre, Manchester M13 9WU, UK; 6 Centre for Genetics and Genomics Versus Arthritis, Centre for Musculoskeletal Research, Faculty of Biology, Medicine and Health, Manchester Academic Health Science Centre, The University of Manchester, Manchester M13 9PT, UK; 7 Division of Rheumatology, University Hospital Frankfurt, Theodor-Stern-Kai 7, 60596 Frankfurt am Main, Germany; 8 TRM Ltd., P.O. Box 493, Scarborough YO11 9FJ, UK; 9 VIB-UGent Center for Inflammation Research, Ghent University, 9052 Ghent, Belgium; 10 Institute of Infection, Immunity and Inflammation, University of Glasgow, Glasgow G12 8QQ, UK; 11 Novartis Pharma AG, CH-4056 Basel, Switzerland; 12 Oxford BioDynamics Limited, Oxford OX4 2JZ, UK; 13 Vital-IT Group, SIB Swiss Institute of Bioinformatics, CH-1015 Lausanne, Switzerland; 14 Fraunhofer IAIS, Institute for Intelligent Analysis and Information Systems, Schloss Birlinghoven 1, 53757 Sankt Augustin, Germany; 15 Department of Development and Regeneration, KU Leuven, Skeletal Biology and Engineering Research Centre, P.O. Box 813 O&N, Herestraat 49, 3000 Leuven, Belgium; 16 Atturos Ltd., c/o UCD Conway Institute, University College Dublin, D04 V1W8 Dublin, Ireland; 17 Glasgow Polyomics, College of Medical, Veterinary and Life Sciences, Garscube Campus, University of Glasgow, Glasgow G61 1QH, UK; 18 UCD Conway Institute, School of Medicine, University College Dublin, Belfield, D04 V1W8 Dublin, Ireland

**Keywords:** psoriatic diseases, psoriatic arthritis, psoriasis, multi-omics, data integration

## Abstract

The definitive diagnosis and early treatment of many immune-mediated inflammatory diseases (IMIDs) is hindered by variable and overlapping clinical manifestations. Psoriatic arthritis (PsA), which develops in ~30% of people with psoriasis, is a key example. This mixed-pattern IMID is apparent in entheseal and synovial musculoskeletal structures, but a definitive diagnosis often can only be made by clinical experts or when an extensive progressive disease state is apparent. As with other IMIDs, the detection of multimodal molecular biomarkers offers some hope for the early diagnosis of PsA and the initiation of effective management and treatment strategies. However, specific biomarkers are not yet available for PsA. The assessment of new markers by genomic and epigenomic profiling, or the analysis of blood and synovial fluid/tissue samples using proteomics, metabolomics and lipidomics, provides hope that complex molecular biomarker profiles could be developed to diagnose PsA. Importantly, the integration of these markers with high-throughput histology, imaging and standardized clinical assessment data provides an important opportunity to develop molecular profiles that could improve the diagnosis of PsA, predict its occurrence in cohorts of individuals with psoriasis, differentiate PsA from other IMIDs, and improve therapeutic responses. In this review, we consider the technologies that are currently deployed in the EU IMI2 project HIPPOCRATES to define biomarker profiles specific for PsA and discuss the advantages of combining multi-omics data to improve the outcome of PsA patients.

## 1. Introduction

### 1.1. Psoriasis and Psoriatic Arthritis

Psoriasis is a chronic, immune-mediated inflammatory disease (IMID) of the skin, which affects 0.91–8.5% of the population, varying by age, region and ethnicity [1]. The most common manifestation is plaque psoriasis (psoriasis vulgaris), which accounts for ~80% of cases and typically involves the formation of erythematous and scaly plaques on the head, ears, elbows and knees, as well as gluteal and umbilical areas. These skin changes are often highly conspicuous, and the resulting stigmatization can lead to psychosocial issues. There is also a high rate of comorbidities, including cardiovascular disease and obesity [2]. Approximately 30% of psoriasis patients go on to develop psoriatic arthritis (PsA) [3], a mixed-pattern IMID characterized by the inflammation of mainly entheseal and synovial musculoskeletal structures [4]. Predisposition to the development of PsA has a strong genetic basis [3] and correlates with the severity of psoriatic skin lesions, including nail involvement (pitting, cracking, separation or nail loss). However, in a minority of cases, the symptoms of PsA develop alongside psoriasis or even before it. Various environmental and lifestyle factors also increase the risk of PsA at the population level, including a high body mass index and smoking [5,6,7], although paradoxically, smoking is negatively associated with progression to PsA at the level of individual psoriasis patients [8]. There is also increasing evidence that dietary factors influence the risk of progressing to PsA [9,10]. PsA can lead to structural damage and loss of function of the joints due to bone erosion, new bone formation and cartilage loss [11]. It has diverse presentations including asymmetric oligo-articular forms of arthritis, polyarticular disease, dactylitis and spinal inflammation [12].

### 1.2. Current Diagnostic Practices and Disease Management Strategies

A diagnosis of psoriasis is usually based on the appearance of the skin [13]. Blood tests or other diagnostic procedures are generally unnecessary [14]. If clinical diagnosis is uncertain, psoriasis can be differentiated from visually similar conditions (such as certain forms of eczema) by skin biopsy, which will confirm epidermal thickening interdigitating with the dermis, changes to the stratum granulosum, the presence of nuclei in the superficial layer, and the presence of infiltrating T cells [15]. In contrast, there is no definitive diagnosis for PsA because the clinical manifestations overlap with other arthritic diseases, including rheumatoid arthritis (RA), osteoarthritis and inflammatory bowel disease (IBD)-associated arthritis. Current diagnostic practice is based on rheumatologic assessment involving physical examination, medical history, blood tests and imaging. More definitive diagnosis is generally dependent on the presence of inflammation and musculoskeletal damage, which makes early intervention much more challenging. The identification of early and specific biomarkers of PsA would facilitate immediate treatment with the most appropriate drugs, therefore offering a much better prognosis for PsA patients and even preventing disease progression in its early stages [16]. In addition to the need for early diagnosis so that treatment can improve patient outcomes, the management of chronic disease plays an important role with a focus on individualized and personalized treatment strategies. Even following the initiation of appropriate immunosuppressive therapy, up to ~40% of patients may not respond or experience adverse effects [17]. There is an urgent medical need for biomarkers that facilitate the early differentiation of PsA and allow the prediction and monitoring of therapeutic responses during the chronic disease stage, thus helping to normalize function and improve outcomes and quality of life.

### 1.3. The Promise of Omics and Multi-Omics Technology

**Figure 1:**
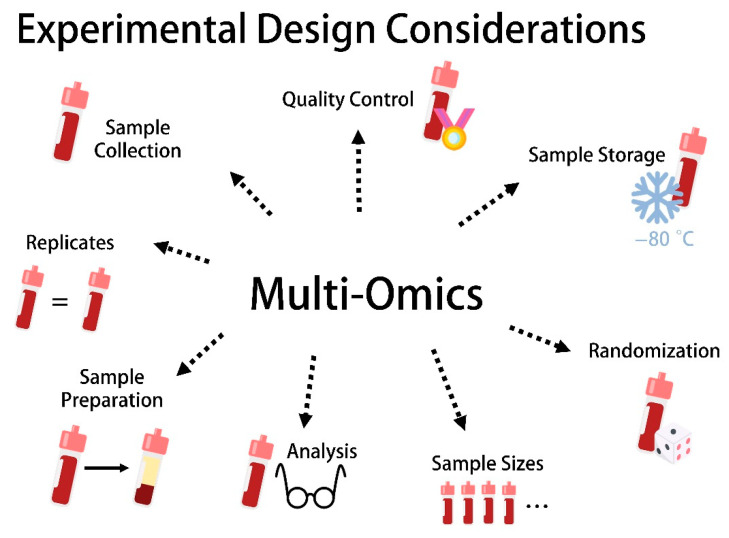
Experimental design considerations for the utilization of multi-omics data.

Biomarkers that are distinct for specific groups of patients can be used for the early diagnosis of diseases because they often correspond to qualitative or even quantitative indicators of biological and pathological processes [18,19]. The genomics revolution in the 2000s identified a large panel of new genetic markers that are associated with particular disease phenotypes, but the potential of biomarkers expanded enormously as omics technology broadened to encompass the global analysis of DNA modifications (epigenomics), RNA (transcriptomics), proteins (proteomics) and metabolites (metabolomics). Furthermore, it is reasonable to differentiate between the analysis of polar metabolites and the analysis of lipids (lipidomics) because the physicochemical properties of these compounds are quite distinct and optimized methods for analyzing these groups are necessary. The advent of proteomics and metabolomics/lipidomics in particular has raised the possibility of using combinations of markers to differentiate between diseases or disease stages in a quantitative manner, which is not possible with genetic markers outside the field of oncology. As the corresponding technologies have become increasingly sophisticated, sensitive and automated, the cost of analysis has fallen and more ambitious studies are possible, including the correlation of multiple omics biomarker profiles across large groups of patients. This requires stringent quality control standards to be applied during sample collection, storage, preparation and analysis, including due attention to sample sizes and replicates, as well as appropriate randomization (Figure 1).

The EU-funded HIPPOCRATES project (https://hippocrates-imi.eu, accessed on 20 September 2022) is an ambitious collaboration that considers the potential of multiple molecular marker types across the spectrum of omics technology and seeks to combine them with conventional clinical diagnostic methods (imaging, medical records and physical examinations) for PsA. The value of omics technologies in the clinical care of PsA patients has been explored in a recent review article, including transcriptomics (which is not part of the HIPPOCRATES project) [20]. HIPPOCRATES aims to extend the concept by combining marker profiles for the differential diagnosis of psoriasis and PsA, as well as prognosis and the monitoring of treatment responses. In this review, we focus on the main objectives of the HIPPOCRATES project by considering the advantages and disadvantages of different omics technologies for the discovery of biomarkers for psoriasis and PsA, the potential of multi-omics approaches that combine different technologies to take advantage of synergies and how the diverse data formats may be combined and interrogated using advanced data evaluation tools (e.g., tools based on artificial intelligence) to identify patterns with diagnostic or prognostic value.

## 2. Genomics

### 2.1. Brief Overview of Relevant Genomics Technologies

Genomics is the branch of biology that deals with the analysis of genomes. In the context of psoriasis and PsA, genomics can be used to identify and characterize the genes, and more importantly the gene variants (alleles), that are associated with each disease. Many of the genes identified as associated with psoriasis have also been found to be associated with PsA when compared to population controls, highlighting their shared genetic basis. Susceptibility loci associated with PsA alone have also been identified, including several *HLA-B* alleles and *IL23R* [21,22]. The detection of pathological gene variants can be used to assist diagnosis and also to predict the age of onset, severity and likely symptoms of the disease. However, the multiple genes that distinguish between psoriasis and PsA may also be shared with other arthritic diseases, such as RA or ankylosing spondylitis.

The fundamental technology underlying the field of genomics is the genome-wide genotyping array, the contents of which are routinely enhanced by imputation, which provides the structure and sequence of key disease-associated genes and allows causative allelic variants to be identified. Genome-wide association studies (GWAS) and gene chip experiments have identified more than 20 additional loci outside the HLA system that are associated with PsA [23,24], some of which are exclusive (i.e., not also associated with psoriasis) [25]. The advent of next-generation sequencing platforms that are faster, cheaper and easier to automate than classic Sanger sequencing will enable researchers to amass a large body of sequence data from various patient cohorts, and this allows the comparison of patient groups to identify relevant alleles, in particular for rare variants not captured on genotyping arrays or by imputation.

### 2.2. Applications for Early Diagnosis, Prognosis and Treatment Monitoring

PsA is known to have a strong genetic component, which means that certain allelic variants are likely to be more prevalent among PsA patients than controls (or other disease cohorts). Because such genetic variation is present from conception, it should be possible to detect disease-causing alleles before the onset of symptoms and commence treatment as early as possible. Similarly, it should be possible to detect PsA-associated alleles in cohorts of psoriasis patients and thus identify those at the greatest risk of progression. Although many different alleles are associated with psoriasis, PsA or both, GWAS can be used to screen for large panels of variants in a single test, which is generally based on array hybridization or multiplex PCR [26,27]. The detection of one or more informative variants can therefore provide data to indicate causality. Other markers may be useful for the assessment of therapy, and to determine which subcomponents are heritable, and therefore more predictable [28]. Accordingly, prospective studies are needed in psoriasis patients, ideally recruited from primary care before disease-modifying therapy commences, to assess the ability of genetic variants to predict the onset of PsA.

### 2.3. Case Studies/Examples in Psoriasis and PsA

The primary genetic factors that distinguish PsA from psoriasis map to the *HLA-B* locus [29,30]. The alleles HLA-B*39, HLA-B*07, HLA-B*38 and in particular HLA-B*27 have been described as specific risk factors for PsA [31]. Although gene mapping is consistent across different studies, resolution to a precise allelic variant is conflicting when the reported index associations point to amino acid positions 45 or 97 (Table 1). Outside the HLA region, there is convincing evidence for a PsA-specific effect at the *IL23R* locus independent of the known psoriasis risk variant [32,33,34]. Other genes associated with PsA but not psoriasis include *KIR2D* [35], *IL4* and *KIF3A* [36], *B3GNT2* [37] and *PTPN22* [25].

## 3. Epigenomics

### 3.1. Brief Overview of Relevant Epigenomics Technologies

Epigenomics is the large-scale analysis of epigenetic phenomena, which include DNA methylation and histone modification as regulators of the 3D configuration of the genome, and the expression of small regulatory RNAs. Epigenetic mechanisms play a key role in the regulation of gene expression, and specific epigenetic markers can be associated with diseases such as psoriasis and PsA. Various technologies can be used to monitor genome-wide epigenetic phenomena, including chromatin immunoprecipitation (ChIP) followed by detection on microarrays (ChIP-chip) [38] or by sequencing (ChIP-Seq) [39], the detection of methylated DNA using bisulfite sequencing or (directly) by nanopore sequencing or SMRT sequencing [40], and enzyme-based chromatin accessibility assays [41]. The detection of chromosome conformation signatures (sequences that are likely to control the 3D structure of the genome) can also be used to pinpoint abnormal chromosome structures that are associated with diseases or responses to treatment. For example, the Oxford Biodynamics EpiSwitch platform is based on the testing of more than 10,000 samples in 30 disease indications, enabling the screening, evaluation, validation and monitoring of 3D genomic biomarkers [42].

### 3.2. Applications for Early Diagnosis, Prognosis and Treatment Monitoring

The EpiSwitch platform facilitates the discovery of stable and heriTable 3D genomic markers and the development of highly sensitive clinical assays based on non-invasive blood readouts. In the case of PsA, it can assist with a definitive diagnosis and prognosis in the context of comorbidities and overlapping symptoms, without resorting to biopsy. This technique has already delivered biomarkers that predict the response to methotrexate treatment in RA patients [43], that predict the response to immune checkpoint inhibitors in cancer [44], and that are prognostic for severe outcomes of COVID-19 based on individual patient immune health profiling [45]. The markers profiled by EpiSwitch technology are governed by all forms of genetic and epigenetic variation, and their combined influence has a major impact on the regulation of gene expression by controlling access to chromatin. Therefore, such markers are powerful high-level integrators of multi-omic signals [46]. In order to utilize the full potential of EpiSwitch, a representative cohort of whole blood samples with clinical annotations is required, representing extreme clinical outcomes. That spectrum will define the quality of the EpiSwitch biomarkers and their correlation with other modalities.

### 3.3. Case Studies/Examples in Psoriasis and PsA

Although chromosome conformation signatures for psoriasis and PsA are not yet available, the promise of the technique has been demonstrated in early RA patients commencing methotrexate treatment [43]. Using blood samples from responders, non-responders and healthy controls, a custom biomarker discovery array was refined to a five-marker chromosome conformation signature that could discriminate between responders and non-responders. Markers were validated using a blinded, independent cohort of 19 early RA patients (9 responders and 10 non-responders) and the corresponding loci were mapped to a RA-specific expression quantitative trait locus (eQTL). Finally, a five-marker chromosome conformation signature was found that could identify, at baseline, responders and non-responders to methotrexate. It consisted of binary chromosome conformations in the genomic regions of *IFNAR1*, *IL-21R*, *IL-23*, *CXCL13* and *IL-17A*. When tested on a cohort of 59 RA patients the marker provided a negative predictive value of 90% for methotrexate response. When tested on a blinded independent validation cohort of 19 early RA patients, the signature demonstrated a true negative response rate of 86%, and 90% sensitivity for the detection of non-responders. Only conformations in responders mapped to the RA-specific eQTL.

## 4. Proteomics

### 4.1. Brief Overview of Relevant Proteomics Technologies

Proteomics can be defined as the large-scale analysis of proteins. In the context of PsA, it has been applied mainly to identify biomarkers that can be detected in blood, synovial fluid or skin samples for the early diagnosis of PsA and its differentiation from psoriasis [47,48,49]. The proteome is much more complex and dynamic than the genome because there are an estimated ~20,000 protein-encoding genes in the human genome [50], but these give rise to multiple variants by alternative transcription, splicing and processing of RNA, post-translational modification and protein–protein interactions. About 10% of the human proteome lacks experimental evidence, and the combined effect of differential protein abundance, protein modifications, sequence variation and interactions further complicate the task of measuring all proteins in every sample [50].

The technologies used to interrogate the proteome can be broadly divided into untargeted methods that attempt to consider all proteins in a sample, and targeted methods that focus on specific proteins or classes of proteins. Mass spectrometry (MS) is a key platform in both approaches because it is a sensitive, high-throughput technology that is relatively easy to automate. Proteins are digested into peptides using a protease with known specificity such as trypsin, and the mass of each peptide, and its fragments generated inside a collision cell, is correlated with values in databases to achieve peptide and hence protein identification. Untargeted methods are based on the analysis of complex, uncharacterized peptide mixtures from multiple proteins. These are generally fractionated by liquid chromatography before injection into the mass spectrometer (LC-MS) and/or by multiple rounds of MS. In the latter case, data-dependent acquisition (DDA) involves the selection of specific peptides during the first round of MS for further fragmentation in subsequent rounds, whereas data-independent acquisition (DIA) involves the fragmentation and further analysis of all peptides from the first round [51]. Targeted methods involve the selection of one or a relatively small number of proteins from a sample for quantitative analysis [52]. Targeted analysis can be undertaken using MS-based methods as exemplified in the Atturos platform or methods that rely on highly specific affinity reagents. In the latter case, the production of high-quality data requires the use of validated binders (affinity reagents) that capture target proteins at low abundance [53]. Current affinity proteomics methods can detect more than 3000 proteins simultaneously by using different selectivity concepts, as well as the amplification capabilities of DNA-based readout methods. One relevant example is the Olink platform, a proximity extension assay that involves the recognition of proteins by antibodies linked to protein-specific DNA barcodes that can be amplified by qPCR or sequencing [54]. This may have a broader dynamic range and greater sensitivity than LC-MS and can simultaneously detect 3000 human proteins in plasma samples [55]. The use of slow off-rate DNA aptamers, provided by SomaLogic, has enabled large-scale studies of 10,000 donors targeting 4000 circulating proteins across human diseases [56].

### 4.2. Applications for Early Diagnosis, Prognosis and Treatment Monitoring

For the proteomic analysis of body fluids, particularly blood, further challenges arise due to the broad concentration range of different proteins, dynamic changes induced by disease processes, and analytical factors that influence protein detection [57]. Collectively, more than 4500 proteins have been detected in plasma samples by discovery-driven MS [58]. Many abundant plasma constituents are secreted by the liver, whereas other secreted proteins, such as inflammatory cytokines, are often elevated only transiently [59]. Accordingly, differences have been observed between individuals and between molecular profiles at longitudinal study time points [60]. When searching for protein biomarkers in healthy individuals, as well as psoriasis and PsA patients, the heterogeneity of signatures from circulating proteins should be expected.

Multiple candidate biomarkers of PsA have been reported in serum and plasma, in addition to a smaller number found in synovial fluid/tissue and skin biopsies [47,48]. Most of the biomarker candidates are proposed for the detection of PsA [61,62], differentiation between mild and severe forms [63,64], measuring disease activity [65], or predicting which psoriasis patients are likely to develop PsA [66]. However, others have been proposed to distinguish PsA from other arthritic diseases such as RA [49,67] or to monitor responses to therapy [68,69,70,71]. For example, the label-free MS analysis of synovial fluid from PsA patients revealed 12 candidate PsA markers including the injury marker MMP3, as well as the inflammatory proteins S100A9 and CRP [62]. A subsequent study using LC-MS identified periostin, which is related to cell-adhesion proteins, and the angiogenesis marker PGK1 [72]. More recently, a systematic search of five bibliographic databases for clinical, laboratory and genetic markers was used to determine the level of evidence for each marker and its association with concomitant/developing PsA [73]. These have been converted into proteomic biomarkers in Table 2. For the prediction of PsA in psoriasis patients, highly characterized cohorts of patients are needed with each disease, minimizing the proportion of undiagnosed subclinical PsA patients in the psoriasis group. Alternatively, longitudinal observation and sample collection in the psoriasis group may directly identify those progressing to PsA, allowing the retrospective analysis of early samples to look for predictive biomarkers.

### 4.3. Case Studies/Examples in Psoriasis and PsA

In a recent study, a set of 951 circulating proteins was analyzed in serum samples to interrogate possible differences between patients with PsA, psoriasis and healthy controls [74]. Sixty-eight differentially expressed proteins were identified when comparing PsA patients and healthy controls, but no differentially expressed proteins were identified when comparing PsA and psoriasis patients. This led the authors to propose a “shared serum proteomic signature” between psoriasis and PsA. However, the cohorts were very small and subclinical PsA in the psoriasis group could not be excluded. Indeed, no information was provided about patient inclusion/exclusion criteria or the criteria used for the differentiation of PsA from psoriasis, which is necessary in such studies. In conclusion, the authors recommended that future studies focus on skin and synovial tissue to find differences between PsA and psoriasis patients.

**Table 2:** Proteomic markers with evidence to support their ability to distinguish between PsA and cutaneous-only psoriasis. A gene-centric table of candidates was created by using the biomarkers listed by Mulder et al. [73]. The proteins and mRNAs were converted into gene-centric entries using the Human Protein Atlas portal (www.proteinatlas.org (accessed on 5 July 2022)), and were annotated for secretion location, tissue expression and biological functional based on the recent clustering of single-cell expression data [75].

Gene Name	Biomarker	UniProt ID	Category	Secretion	Tissue Expression	Biological Function
*ADIPOQ*	Adiponectin	Q15848	Lipid	Blood	Adipose tissue	ECM organization
*APOA1*	ApoA	P02467	Lipid	Blood	Liver	Metabolism
*APOB*	ApoB	P04114	Lipid	Blood	Liver	Metabolism
*CMC2*	C16ORF61	Q9NRP2	Skin	N/A	Non-specific	Mitochondria
*COL2A1*	C2C	P02458	Bone	ECM	Epididymis	Unknown function
*CCL1*	CCL1	P22362	mRNA	Blood	T cells	Adaptive immune response
*CCL20*	CCL20	P78556	mRNA	Blood	Smooth muscle tissue	Mixed function
*CCL7*	CCL7	P80098	mRNA	Blood	Neutrophils	Humoral immune response
*CD5L*	CD5L	O43866	Serum	Blood	Macrophages	Immune response
*COMP*	COMP	P49747	Bone	ECM	Skin	Epidermis development
*C9*	Complement C9	P02748	Serum	Blood	Liver	Hemostasis and lipid
*COL2A1*	CPII	P02458	Bone	ECM	Epididymis	Unknown function
*CPN2*	CPN2	P22792	Skin	Blood	Liver	Hemostasis
*CRP*	CRP	P02741	Inflammation	Blood	Liver	Hemostasis
*COL1A1*	CTX	P02452	Bone	ECM	Fibroblasts	ECM organization
*CX3CL1*	CX3CL1	P78423	mRNA	Blood	Adipose tissue	ECM organization
*CXCL10*	CXCL10	P02778	Cytokines	Blood	Immune cells	Immune response
*CXCL12*	CXCL12	P48061	Skin	Blood	Fibroblasts	ECM organization
*CXCL2*	CXCL2	P19875	mRNA	Blood	Liver	Metabolism
*CXCL5*	CXCL5	P42830	mRNA	Blood	Salivary gland	Salivary secretion
*DKK1*	DKK-1	O94907	Bone	Other	Adipose tissue	ECM organization
*ESR1*	ESR	P03372	Inflammation	N/A	Fibroblasts	ECM organization
*FHL1*	FHL1	Q13642	Skin	N/A	Striated muscle	Muscle contraction
*GSN*	Gelsolin	P06396	Serum	Blood	Fibroblasts	ECM organization
*GPS1*	GPS1	Q13098	Skin	N/A	Non-specific	Mitochondria
*HAT1*	HAT1	O14929	mRNA	N/A	Non-specific	Ribosome
*IFI16*	IFI16	Q16666	Serum	N/A	Immune cells	Immune response
*IL12A*	IL-12/23 p40	P29459	Cytokines	Blood	Brain and skin	Unknown function
*IL12B*	IL-12/23 p40	P29460	Cytokines	Blood	Non-specific	Cell cycle regulation
*IL9*	IL-12/23 p40	P15248	Cytokines	Blood	N/A	N/A
*IL17A*	IL-17	Q16552	Cell culture secretion	Blood	Immune cells	Immune response
*IL17C*	IL-17C	Q9P0M4	mRNA	Blood	Testis	DNA repair
*IL17F*	IL-17F	Q96PD4	mRNA	Blood	B cells	Humoral immune response
*IL2*	IL-2	P60568	Cell culture secretion	Blood	N/A	N/A
*IL23*	IL-23	Q9NPF7	Cytokines	Blood	B cells	Humoral immune response
*IL23R*	IL23R	Q5VWK5	Skin	N/A	Intestine	Brush border
*IL3*	IL-3	P08700	mRNA	Blood	N/A	N/A
*IL33*	IL-33	O95760	Cytokines	Blood	Fibroblasts	ECM organization
*IL34*	IL-34	Q6ZMJ4	Cytokines	Blood	Macrophages	Immune response
*EBI3*	IL-35	Q14213	Cytokines	Blood	Placenta	Pregnancy
*IL12A*	IL-35	P29459	Cytokines	Blood	Brain and skin	Unknown function
*IL36A*	IL-36a	Q9UHA7	Cytokines	Blood	Esophagus	Epithelial cell function
*IL1F10*	IL-38	Q8WWZ1	Cytokines	Blood	Skin	Cornification
*IL6*	IL-6	P05231	Cytokines, mRNA	Blood	Adipose tissue	ECM organization
*CXCL8*	IL-8	P10145	mRNA	Blood	Neutrophils	Humoral immune response
*INS*	Insulin	P01308	Lipid	Blood	Pancreas	Digestion
*ISG20*	ISG20	Q96AZ6	mRNA	N/A	Immune cells	Immune response
*ITGB5*	ITGB5	P18084	Serum	N/A	Adipose tissue	ECM organization
*ITGB5*	ITGB5	P18084	Skin	N/A	Adipose tissue	ECM organization
*KRT17*	K17	Q04695	Serum	N/A	Skin	Epidermis development
*LEP*	Leptin	P41159	Lipid	Blood	Adipose tissue	ECM organization
*LGALS3BP*	M2BP	Q08380	Serum	Blood	Stomach	Digestion
*CSF1*	M-CSF	P09603	Cytokines	Blood	Non-specific	Angiogenesis
*MMP3*	MMP3	P08254	Bone, mRNA	ECM	Salivary gland	Salivary secretion
*MPO*	MPO	P05164	Serum	Membrane	Neutrophils	Humoral immune response
*NOTCH2NLA*	NOTCH2NL	Q7Z3S9	mRNA	Blood	Testis	DNA repair
*TNFRSF11B*	OPG	O00300	Bone	Other	Kidney	Transmembrane transport
*POSTN*	POSTN	Q15063	Skin	ECM	Skin	Epidermis development
*PTPA*	PPP2R4	Q15257	Skin	N/A	Non-specific	Mitochondria
*PRL*	PRL	P01236	Serum	Blood	Pituitary gland	Hormone signaling
*TNFSF11*	RANKL	O14788	Bone	Blood	Immune cells	Immune response
*SETD2*	SETD2	Q9BYW2	mRNA	N/A	Non-specific	Transcription
*IL2RA*	sIL2R	P01589	Serum	N/A	Immune cells	Immune response
*IL2RB*	sIL2R	P14784	Serum	N/A	Immune cells	Immune response
*IL2RG*	sIL2R	P31785	Serum	N/A	T cells	Adaptive immune response
*SNCA*	SNCA	P37840	Skin	Membrane	Brain and bone marrow	Chromatin organization
*SRP14*	SRP14	P37108	Skin	N/A	Non-specific	Mitochondria
*SRPX*	SRPX	P78539	Skin	Unknown	Adipose tissue	ECM organization
*STAT3*	STAT3	P40763	mRNA	N/A	Non-specific	Mitochondria and proteasome
*STAT6*	STAT6	P42226	mRNA	N/A	Macrophages	Immune response
*STIP1*	STIP1	P31948	Serum	N/A	Non-specific	Unknown function
*SYK*	SYK	P43405	mRNA	N/A	Non-specific	Transcription
*TBX21*	TBX21	Q9UL17	mRNA	N/A	Immune cells	Immune response
*TNF*	TNF-alpha	P01375	Cytokines	Blood	Neutrophils	Inflammatory response
*VCP*	VCP	P55072	Serum	N/A	Non-specific	Mitochondria
*FLT4*	VEGFR-3	P35916	Serum	Blood	Non-specific	Transcription
*CHI3L1*	YKL-40	P36222	Serum	Blood	Liver	Metabolism

## 5. Metabolomics

### 5.1. Brief Overview of Relevant Metabolomics Technologies

Metabolomics can be defined as the investigation of changes in the populations of endogenous and exogenous low-molecular-weight metabolites (<1500 Da), representing a shift from single metabolite monitoring to complex profiling and pattern recognition [76]. This is a considerable analytical challenge that involves the identification and quantification of a broad spectrum of molecules in biological matrices such as human plasma or urine, which contain hundreds or thousands of metabolites with diverse chemical and physical properties across a wide dynamic range of concentrations. The most widely used techniques include nuclear magnetic resonance (NMR) spectroscopy and mass spectrometry in combination with gas chromatography (GC-MS) or liquid chromatography (LC-MS). Advanced bioinformatics and statistical tools are used to maximize the recovery of information from the resulting metabolomic datasets.

### 5.2. Applications for Early Diagnosis, Prognosis and Treatment Monitoring

Low-molecular-weight metabolites are important indicators and even integrators of phenotypes, reflecting the biochemical activity of cells and tissues. Metabolomics recognizes that changes in cell function are most evident at the level of small-molecule metabolism and can provide a coherent view of the response of individuals to a variety of genetic and environmental influences [77]. The abnormal cellular processes associated with disease often disrupt the composition of low-molecular-weight metabolites. Perturbations in metabolite abundance and temporal profiles in readily accessible body fluids may provide an index of disease severity through the direct measurement of biochemical changes. As such, metabolomics has the potential to identify biomarkers of PsA that may improve diagnostic accuracy and predict disease progression as well as defining patient responses to specific therapeutic interventions. Similarly, metabolomics may offer additional insight into the metabolic pathways that drive the chronic, immune-mediated processes that are characteristic of PsA, opening routes to potential new drug targets.

### 5.3. Case Studies/Examples in Psoriasis and PsA

Researchers are increasingly using metabolomics for the clinical assessment of PsA [78,79,80]. Several studies have reported alterations in the metabolomes of PsA patients in comparison to healthy controls or individuals with related inflammatory diseases such as psoriasis or RA. The serum levels of various amino acids are modified in PsA patients relative to RA cohorts [81,82]. Changes in the levels of circulating glucuronic acid and α-ketoglutaric acid were detected among psoriasis patients with or without PsA [83] and a correlation was made between serum levels of the choline metabolite trimethylamine *N*-oxide (TMAO) and inflammation in PsA patients [84]. A more recent study used untargeted metabolomics to characterize the metabolic changes in the transition from psoriasis to PsA, revealing differences in the abundance of bile acids (particularly glycoursodeoxycholic acid sulfate) and butyrate to differentiate between psoriasis patients who did or did not progress to PsA [85].

Metabolite profiles in other matrices can also provide a window of opportunity to elucidate the metabolic changes in PsA. It was recently reported that α/β-turmerone, glycerol 1-hexadecanoate, dihydrosphingosine, pantothenic acid and glutamine may act as fecal biomarkers for PsA [86]. In addition, a metabolomic study focusing on urinary metabolites revealed lower levels of citrate, alanine, methylsuccinate and trigonelline in PsA patients compared to unaffected individuals [87]. Metabolomic approaches have also been used to evaluate PsA patient responses to anti-TNF therapy. For example, histamine, glutamine, phenylacetic acid, xanthine, xanthurenic acid and creatinine levels were elevated in urine samples from patients who responded to TNF antagonists, whereas ethanolamine, *p*-hydroxyphenylpyruvic acid and phosphocreatine levels were depleted [88].

## 6. Lipidomics

### 6.1. Brief Overview of Relevant Lipidomics Technologies

Recent technological improvements in LC-MS enable comprehensive lipidomic analysis in clinical studies, analyzing extensive sample sets for different lipids and lipid mediators. Depending on the specific lipids, targeted or untargeted LC-MS may be most appropriate. The untargeted approach is based on high-resolution mass spectrometry (HRMS) and can potentially examine the whole lipidome in a single run, but focuses on the more abundant lipids because the dynamic range is not sufficient to detect scarce molecules such as lipid mediators alongside abundant lipids such as triglycerides. Scarce lipids are analyzed using targeted approaches based on tandem mass spectrometry (MS/MS), which has greater sensitivity and selectivity. However, targeted methods cannot display the whole lipidome, so an approach combining untargeted and targeted methods is used for comprehensive lipidomics analysis, searching for lipids and lipid mediators relevant in the context of psoriatic diseases.

### 6.2. Applications for Early Diagnosis, Prognosis and Treatment Monitoring

Lipids and lipid mediators play a fundamental role in the immune system and changes in homeostatic status are closely related to IMIDs [89,90,91,92,93] such as RA [94], IBD [95,96,97,98,99] and psoriatic diseases [100,101,102,103]. Lipids are involved in many different processes and are also essential building blocks of membranes and key components in energy metabolism. Lipid mediators such as oxylipins and endocannabinoids, which are present at very low concentrations, are signaling molecules implicated in diverse physiological and pathological processes. Therefore, lipid profiles might be used as biomarkers for early diagnosis, prognosis of disease progression or the development of comorbidities, and to guide the selection of the most promising therapeutic approach.

Biomarker discovery in the field of lipidomics is challenging due to strict procedural requirements for sampling, sample preparation and analysis. This is partly due to the variable concentration of lipids in different biological matrices, the broad spectrum of isomeric compounds and the special procedures required to ensure lipid stability at all stages, including pre-analytical sample handling. On the other hand, lipidomics covers a field of up to several thousand different molecules and one of its key advantages is the close temporal linkage between these markers and individual clinical phenotypes or disease states [77,104].

### 6.3. Case Studies/Examples in Psoriasis and PsA

The close link between lipid profiles and IMIDs was recently demonstrated in PsA patients, where oxylipins [102,103,105,106], endocannabinoids [103,107], fatty acids [105,106,107] and phospholipids [106] were found to be potentially pathophysiologically relevant. A recent study also found that the level of inflammatory lipid mediators in psoriasis patients increased following a PsA diagnosis, particularly leukotriene B4 [85]. However, a comprehensive study is required to compare the lipid profiles of patients with psoriasis and PsA, and this will be the first step toward the identification of lipid biomarkers that improve the diagnosis and treatment of PsA.

## 7. Complementary Technologies—Multiple Sequential Immunohistochemistry

Several multiple immunohistochemistry systems have been developed that allow the staining of tissue sections with a theoretically unlimited number of antibodies. The technology makes use of directly labeled antibodies carrying a fluorophore or heavy metal ion. The antibodies are applied to the sample in an automated process, which includes a short incubation period, washing steps, imaging and signal removal. The latter involves either bleaching or chemical inactivation, and is followed by the addition of the next antibody [108,109]. This process can be repeated as often as necessary, and typically creates image stacks representing 30–50 antibodies. Recent developments include software that combines single-cell phenotyping and localized information about neighboring cells, facilitating a quantitative “tissue FACS analysis” (FACS = fluorescence-activated cell sorting) with the description of disease-specific immunological neighborhoods within inflamed tissues [108].

One of the key benefits of multiple immunohistochemistry systems in the context of PsA is single-cell phenotyping in different patient groups using 40–50 antibody probes in automated cycles. By detecting and quantifying a large panel of corresponding markers, it is possible to identify nearly all immune cells and their subtypes, and to characterize their cellular neighborhood to quantify and visualize cellular networks (information that is lost during FACS analysis). The comparison of samples from psoriasis and PsA cohorts can therefore identify differences between the patient groups and generate information about biomarkers and immune cell networks/interactions that may lead to new therapeutic options.

## 8. Data Management/Integration and Artificial Intelligence

To benefit from the wealth of methods used to mine multi-omics data, it is essential to align the data and verify their quality before integration. Data should be formatted according to international standards, including standard bioinformatics file formats (such as FASTA, FASTQ, SAM/BAM, VCF and GFF), and “minimum information” standards for omics experiments [110], including MIGS/MIMS for genomics [111] and MIAPE for proteomics [112]. The data must be checked to ensure they include the same annotation references (e.g., genome version, standard gene and protein names). This is challenging with lipidomics and metabolomics data where there are currently no widely accepted standards, although efforts are ongoing to establish equivalent minimum information standard such as MIAMET [113] as well as standards for lipidomics analysis [114,115]. Following de-identification, clinical data are standardized using the OMOP common data model (Observational Health Data Sciences and Informatics, OHDSI program, available at https://ohdsi.org/ (accessed on 20 June 2022) and aligned to standard dictionaries to ensure interoperability. Once formatted and standardized, data are stored and accessed via a secure data management infrastructure specifically designed to protect sensitive clinical and biomedical data [116].

Multiple processing steps should be considered to ensure data integrity, including missing value imputation, normalization, transformations, aggregation and batch effect correction [117,118,119]. Unsupervised multivariate analysis methods such as common dimensions [120] can be used to assess overall variability, trends and potential biases across multiple integrated layers of multi-omics and clinical data before further data exploration by supervised multivariate analysis methods such as OPLS-DA [121] or artificial intelligence-based approaches such as machine learning. A wide range of computational methods can be applied depending on the study design and research aim. In addition to classical statistical analysis, machine learning can be used to evaluate data in an unsupervised manner for preliminary exploration and dimensional reduction (e.g., clustering approaches such as DBSCAN or k-means algorithms, or dimensional reduction such as PCA or TSNE). Batch effects in dimensional reduction and clustering approaches can reveal outliers [118]. In a clinical setting, supervised machine learning often tackles classification problems rather than regression. Due to the high dimensionality of multi-omics data and the so-called “curse of dimensionality” (low number of subjects and high number of features), feature selection algorithms such as LASSO or ridge can be applied to enhance the results of supervised learning [118,122]. In addition to feature selection, class imbalances are common challenges in multi-omics analysis, but can be solved by sampling or cost-sensitive learning [122]. Commonly used algorithms such as random forests, support vector machines and the k-nearest neighbor algorithm can provide insight into the underlying structures of datasets and can be developed into powerful models for the support of clinical decision making [123,124,125]. In order to develop further hypotheses and integrate data with the literature, pathway analysis can embed the data in a broader context [126].

## 9. The Advantage of Multi-Omics Evaluation

As discussed above, several markers have been identified that commonly occur in PsA patients, but no single marker stands alone as a specific indicator of the disease. Even the most reliable markers are also present in other IMIDs, which therefore makes it difficult if not impossible to achieve a definitive diagnosis. In other fields, the lack of definitive qualitative markers has been addressed by (a) seeking quantitative markers, whose abundance rather than presence/absence correlates with a disease, and (b) profiles based on combinations of several markers that are more informative than single molecules, a strategy first applied to ovarian cancer [127]. Indeed, this approach has also been successful in RA, where the proteomic analysis of serum and synovial fluid has revealed the elevation of multiple biomarkers representing processes such as joint inflammation and injury (e.g., MMP3, IL-12, IL-15 and IL-18), cartilage integrity and bone or connective tissue degradation (e.g., MMP13 and neoepitopes of collagen) [128]. Considering that such panels of RA markers have been assembled based solely on proteomics data, it is clear that the combination of proteomics with orthogonal omics datasets plus more diverse data can increase the power of this approach exponentially, both for diagnosis/prognosis [129] and the monitoring of drug responses [130]. In one recent study, metabolomics and lipidomics analysis revealed that a combination of the bile acid conjugate glycoursodeoxycholic acid sulfate and lipid mediator leukotriene B4 provided a sensitive and specific predictor of progression from psoriasis to PsA [85]. However, adding new features will also require the careful evaluation of added value, both for discovery in basic research and translation to the clinic. Models that incorporate more markers may be more sensitive and specific, but the cost of acquiring the data in routine clinical practice may be prohibitive, although this may not always be the case [131]. A risk remains that expensive and large datasets merely report already known aspects, such as the effect of the body mass index or inflammation on disease progression. The field should also strive to identify causal markers rather than solely correlative observations without a direct biochemical connection to the phenotype. As the amount and complexity of the data increase, it becomes more difficult for humans to identify consistent patterns that correlate with certain diseases, but machine learning algorithms either supervised to assign samples to known categories or devising categories de novo using unsupervised analysis have the power to reveal these hidden patterns and then to apply the same approach when analyzing data from new patients, greatly improving the accuracy of the resulting predictions.

## 10. Conclusions and Outlook

The definitive diagnosis and early treatment of PsA are not yet possible because the clinical manifestations and associated biomarkers are not, on an individual basis, able to distinguish PsA from other IMIDs or predict those individuals with psoriasis who will progress to PsA. However, the combination of biomarker profiles based on data from multi-omics technologies and classical sources, such as imaging data and clinical evaluations, could provide the body of information required for early diagnosis and the initiation of effective treatment before symptoms emerge. Combinations of different types of biomarkers have proven effective in other fields, particularly oncology, but such biomarker profiles are often mainly based on a single method. The power of biomarker profiles may increase with the number of complementary modalities that can be tested simultaneously. For PsA, combining information from disease-associated alleles and chromatin structures, the levels of proteins, lipids and other metabolites, multimodal image analysis, histology and classical phenotyping will provide an important step forward. Ultimately, the use of multiple orthogonal technologies that embed machine learning will lead to the generation of unique molecular and clinical fingerprints that can be used for PsA diagnosis, prognosis and therapeutic monitoring. However, research on the identification of biomarker profiles/fingerprints using different omics technologies is still in the discovery phase with much work to be conducted to turn the anticipated results of these analyses into assays which are applicable in routine clinical settings. The HIPPOCRATES project is therefore strategically important because it combines expertise from all relevant fields with access to comprehensive cohorts, technologies and translational research experience. This will ultimately improve the quality of life for those living with PsA or at risk of developing PsA.

## Figures and Tables

**Table 1:** Genetic variants with evidence to support their ability to distinguish between PsA and cutaneous-only psoriasis.

Chromosome	Gene or Locus	Variant ID	Ref.
6	*HLA-B*	Amino acid position 45	[30]
6	*HLA-B*	Amino acid position 97	[29]
1	*IL23R*	rs12044149	[32,33,34]
5	*5q31 (IL4, KIF3A)*	rs715285	[36]
1	*PTPN22*	rs2476601	[25]
6	*TNFAIP3*	rs9321623	[34]
19	*KIR2D*		[35]
2	*B3GNT2*		[37]

## Data Availability

Not applicable.
